# Compounding asymmetries in nucleic acid synthesis screening: policy pathways for strengthening global biosecurity governance

**DOI:** 10.3389/fbioe.2026.1819575

**Published:** 2026-06-10

**Authors:** Sana Zakaria, Sophie Peresson, Curtis Matthew Sharkey, Kirsten Engel

**Affiliations:** 1 RAND Europe, Cambridge, United Kingdom; 2 The International Biosecurity and Biosafety Initiative for Science, Geneva, Switzerland; 3 RAND Corporation, Santa Monica, CA, United States; 4 SecureDNA, Basel, Switzerland

**Keywords:** biosecurity, global security, governance, screening, swiss cheese model (SCM)

## Abstract

Order screening for synthetic nucleic acids has become a central element of biosecurity policy in several jurisdictions, including the United States, European Union, New Zealand, and United Kingdom. Yet global uptake remains uneven. Many providers, particularly in low- and middle-income countries (LMICs), operate without explicit screening mandates, often because national policy frameworks have not yet engaged with synthesis focussed biosecurity rather than because screening is actively opposed. Data from the IBBIS Global DNA Synthesis Map indicate that only a small fraction of more than 700 known providers worldwide maintain publicly identifiable screening mechanisms, with policy mandates and operational infrastructure absent simultaneously in many regions. This perspective argues that narrowing global screening asymmetries requires reframing adoption as a spectrum rather than an all-or-nothing compliance threshold. We analyse how technical, economic, and governance disparities compound over time, examine how advances in AI-enabled biological design intensify these dynamics, and outline stepwise policy strategies to lower entry barriers, expand participation, and promote more equitable global biosecurity governance. We situate synthesis screening within a layered defence framework in which multiple, imperfect but complementary safeguards reduce systemic risk when broadly and consistently adopted.

## 1 Introduction

Over the past 2 decades, biotechnology has transformed through rapid advances in DNA synthesis, sequencing, and assembly technologies, reducing costs and increasing accessibility. The global DNA synthesis market, valued at $5.95 billion in 2026, is projected to reach $24.06 billion by 2034, growing at a compound annual rate of 19.09%, driven by demand for custom gene synthesis in research, therapeutics, and agriculture. The commercialisation of synthetic nucleic acids, has shifted supply chains from specialised institutions to a global network of providers, enabling academics, industries, and even community labs to order complex genetic constructs with ease (Fortune Business Insights, 2026; National Academies of Sciences Engineering and Medicine, 2018).

Parallel to advances in DNA synthesis, artificial intelligence (AI)-enabled biological design tools are reshaping the ideation and prototyping phases of biological research by automating tasks that previously required substantial expert time and iterative experimentation. Deep learning systems for protein structure prediction, most prominently AlphaFold, now achieve near-experimental accuracy across large swathes of the proteome (Jumper, et al., 2021; Varadi, et al., 2024), enabling researchers to infer structure–function relationships and prioritise designs without extensive wet-lab screening. Generative sequence design and foundation models support the *de novo* creation of functional biomolecules, including enzymes and therapeutic proteins, and are beginning to extend from single proteins to higher-order assemblies (Krapp, et al., 2024; Zhang, et al., 2025). Recent work using the Evo family of genome-scale models has experimentally demonstrated AI-guided design of diverse, viable bacteriophage genomes (King, et al., 2025), illustrating how learned sequence–function mappings can be leveraged to generate complex genetic systems. Collectively, these tools compress design–build–test cycles, reduce dependence on highly specialised expertise, and broaden the set of actors able to carry out sophisticated biological design, thereby accelerating innovation while introducing new vectors for misuse in a more democratised biotechnology landscape (National Academies of Sciences Engineering and Medicine, 2025). Together, these developments have amplified both the scale and accessibility of synthetic biology. In doing so, they have elevated nucleic acid synthesis to a critical governance chokepoint: the same materials that enable beneficial innovation also enable the *de novo* construction, modification, or enhancement of potentially harmful biological agents.

As access expands across jurisdictions with differing regulatory capacities, uneven adoption of screening measures creates structural asymmetries in biosecurity oversight. These asymmetries are not merely administrative; they shape deterrence dynamics, market incentives, and global risk distribution. We frame this landscape using a Swiss cheese model of biosecurity, in which nucleic acid synthesis screening sits alongside other layers, including customer verification, export control licensing, institutional biosafety governance, and downstream surveillance, as one slice in a multi-layered defence. Each layer has intrinsic limitations and “holes,” but when widely and consistently adopted they can collectively raise the probability that suspicious activity is detected and disrupted somewhere in the system.

We take a high-level view of the gene synthesis industry’s development, challenges facing maintaining security in the distribution of certain hazardous synthetic genetic materials, and consider whether asymmetries in the gene synthesis security landscape are developing and how those asymmetries can be minimized, moving forward. This perspective draws on published policy documents, international standards (including the ISO 20688 series), publicly available data from the IBBIS Global DNA Synthesis Map, and the Swiss cheese conceptual framework to analyse compounding asymmetries and identify policy pathways for narrowing them. We first characterise the current screening dichotomy across jurisdictions (Section 2), then examine how asymmetries compound through reinforcing technical, economic, governance, and AI-related feedback loops (Section 3). We outline a spectrum-based policy approach to expanding participation (Section 4) and conclude with a discussion of implications and limitations (Section 5).

## 2 The screening dichotomy: adopters, partial adopters and non-adopters

Over the past decade, nucleic acid synthesis screening has evolved from a voluntary industry norm into an emerging policy expectation in a subset of jurisdictions. Yet global adoption remains uneven. Jurisdictions can be broadly categorised into adopters, which have formalised screening expectations through regulatory, legislative, or binding policy instruments; partial adopters, which rely primarily on guidance, voluntary standards, or fragmented oversight; and non-adopters, where no explicit screening frameworks exist.

At the international level, industry-led initiatives such as the International Gene Synthesis Consortium (IGSC) have long maintained voluntary screening commitments. A significant milestone occurred with the publication of ISO 20688-2:2024, the first globally negotiated technical standard specifying requirements for screening synthetic nucleic acid orders (IGSC, 2024; ISO, 2024). This standard sits within the broader ISO 20688 series: Part 1 (ISO, 2020) addresses oligonucleotide production and quality control for sequences up to approximately 250 bases, while Part 2 covers longer gene fragments, genes, and genomes and incorporates biosecurity screening provisions. Both standards were developed under ISO Technical Committee 276 (Biotechnology), signalling increasing international codification of screening expectations, though neither mandates national adoption.

Among adopters, the United States has progressively linked screening expectations to federal funding structures. The May 2025 Executive Order on “Improving the Safety and Security of Biological Research” directs federal agencies to develop a strategy for achieving “comprehensive, scalable, and verifiable nucleic acid synthesis screening,” extending beyond federally funded research contexts (EOP, 2025). Additionally, Congress has recently begun considering legislation mandating sequence and customer screening (Cotton and Klobuchar, 2026; Salinas and McCormick, 2025). The European Union has signalled harmonisation through the proposed European Biotech Act (December 2025), which includes binding screening obligations, suspicious-transaction reporting mechanisms, and requirements that benchtop synthesis devices incorporate screening functions (European Commission, 2025; Pour, 2026). New Zealand’s Gene Technology Bill proposes legislating screening directly within a broader gene technology reform package, including approval and oversight of providers, offences for non-compliance, and regulation-making powers covering sequence screening, customer screening, and benchtop equipment (Health Committee, 2025).

The United Kingdom represents a partial adopter model grounded in operational guidance rather than statute. The 2024 Screening Guidance establishes baseline expectations for customer screening, sequence screening, escalation procedures, and extension to benchtop synthesis equipment, though compliance remains voluntary (DSIT, 2024).

In contrast, large segments of provider markets across Asia, the Middle East, Africa, and parts of Latin America operate without explicit screening mandates. Data from IBBIS indicate that only 69 of more than 700 known providers globally have publicly identifiable screening mechanisms (IBBIS, 2025a). IBBIS’s Africa-focused mapping, involving in-depth interviews across 15 countries, reveals a consistent pattern: researchers reported little to no screening from the providers they used, ‘largely because few African countries have clear regulations around DNA synthesis’ (IBBIS, 2025a; IBBIS, 2025b). Non-adoption often reflects not opposition but the absence of policy frameworks, cost constraints, and limited regulatory clarity.

Disparities in adoption tend to compound over time. Jurisdictions that institutionalise screening develop technical expertise, benchmarking tools, compliance ecosystems, and shared standards. Non-adopters face increasing relative barriers as expectations evolve. In Swiss cheese terms, regulatory arbitrage effectively aligns the holes across layers: actors can exploit absent screening or weak customer checks in specific jurisdictions, so that protection is determined not by best practice but by the least stringent combination available. As participation broadens, deterrence effects increase disproportionately; conversely, systemic risk remains anchored to the least stringent nodes.

## 3 Asymmetries in screening adoption could compound over time

Asymmetries in nucleic acid synthesis screening do not remain static. Differences in adoption and capacity tend to widen as technical, regulatory, and commercial dynamics reinforce one another. Early adopters accumulate experience, infrastructure, and institutional familiarity that support incremental refinement while late adopters face rising relative barriers to entry as expectations evolve.

### 3.1 Technical feedback loops

Providers in early-adopting jurisdictions are moving beyond basic taxonomy- or homology-based screening toward more sophisticated approaches, including function- or phenotype-oriented flags, curated and benchmarked sequence-of-concern datasets, and multi-layered Know-Your-Customer (KYC) and Know-Your-Business (KYB) protocols. These developments are reflected in evolving frameworks such as the U.S. Screening Framework Guidance (ASPR, 2023a; ASPR, 2023b), the UK Screening Guidance (DSIT, 2024), and ISO 20688-2:2024 (ISO, 2024). Recent work by the Sequence Biosecurity Research Consortium (SBRC) to develop standardised definitions for sequences of concern represents a further step toward interoperable technical baselines (Beal et al., 2026; SBRC, 2025). Additionally, there have been recent calls for the implementation of a KYC approach to managing tiered access to highly capable AI-enabled biological tools (Carter and Butchello, 2026). So, it is likely that as the interface between AI and synthetic biology becomes increasingly hazardous these approaches will become increasingly important. While these second-generation practices improve risk discrimination, they remain constrained by false positives and negatives from incomplete reference datasets, reliance on length thresholds, and well-documented evasion strategies such as order fragmentation and codon recoding (National Academies of Sciences Engineering and Medicine, 2023; Diggans and Leproust, 2019; Carter et al., 2021).

### 3.2 Economic feedback loops

Providers implementing limited or no screening may realise short-term commercial advantages through lower prices, faster turnaround, and fewer administrative requirements, attracting customers whose orders might trigger scrutiny elsewhere. This dynamic creates a classic free-rider problem: responsible providers internalise compliance costs while the collective protective value of screening remains constrained by the least stringent nodes in the supply chain. Recent cost–benefit analysis of synthesis screening in the UK and the EU demonstrates that the economic case for screening can be favourable even when accounting for provider and researcher concerns, and that the costs of inaction, measured in potential public health and security impacts, substantially exceed implementation costs (Zakaria et al., 2026; Fady et al., 2025). Nevertheless, absent mechanisms to socialise compliance costs or reward responsible practice, market selection pressures risk entrenching a two-tier system in which incentives to invest in screening are unevenly distributed, limiting the deterrent effect that broader participation could generate.

### 3.3 Governance divergence and interoperability gaps

Divergent definitions of “risk” and “sequences of concern” further amplify asymmetries. Providers and regulators employ varying blacklists, length thresholds, contextual criteria, and escalation rules, producing inconsistent outcomes for similar orders. This lack of interoperability complicates benchmarking, cross-border information sharing, and coordinated responses to suspicious activity. Emerging multi-stakeholder initiatives including IBBIS’s DNA Screening Standards Consortium (DSSC), the SBRC, and recent proposals for safe-harbour information sharing (Seltzer et al., 2026), seek to harmonise definitions and establish minimum expectations, but participation remains voluntary and uneven. For LMIC providers, governance fragmentation introduces an additional deterrent: faced with multiple overlapping frameworks, providers without in-house biosecurity expertise may find it difficult to identify an actionable starting point. IBBIS’s Common Mechanism was specifically conceived to address this: developed with experts from Africa, Asia, Europe, and North America, and hosted in Switzerland to avoid perception of alignment with any single national system (IBBIS, 2024; NTI, 2024).

### 3.4 AI-enabled capability divergence

Advances in AI-assisted biological design are likely to intensify these dynamics, though the nature of the risk varies significantly across AI tool categories. Protein structure prediction tools such as AlphaFold primarily lower barriers to understanding existing biology. Generative sequence design models can produce novel functional sequences with reduced homology to known threats, posing more direct challenges to identity-based screening. Genome-scale foundation models such as the Evo family can design complex multi-component genetic systems, representing a qualitatively different capability (King et al., 2025; Callaway, 2026). These categories pose distinct challenges for screening systems and require differentiated governance responses.

Recent studies demonstrate that machine learning can generate protein sequences preserving function while evading homology-based detection, prompting updates to screening methodologies by major providers (Wittmann et al., 2025; Abel et al., 2026). However, subsequent stress-testing of updated screening software against fragmented AI-redesigned sequences reveals that performance varies across tools and that fragment-level detection remains a significant challenge (Wittmann et al., 2026). The vulnerability is not merely theoretical: a red-team study demonstrated that 36 of 38 DNA synthesis providers, including 12 of 13 IGSC members, shipped fragments sufficient to reconstruct the 1918 influenza genome when ordered by a fictitious organisation with no laboratory (Edison et al., 2026). This underscores that current screening can be bypassed through fragmentation across providers. Recent work has begun developing approaches to short-fragment screening that consider context across orders rather than screening sequences in isolation (Gemler et al., 2026), but these capabilities remain nascent.

KYC provisions further illustrate compounding effects. In early-adopting jurisdictions, guidance encourages assessment of customer legitimacy, yet criteria often remain qualitative and open to interpretation. Emerging frameworks such as the “Know Your Scientist” model, which draws on anti-money laundering principles to shift governance from content inspection to user verification (Feldman et al., 2026), offer promising architectural alternatives but remain early-stage. Without deliberate strategies to lower entry barriers and harmonise baseline expectations, asymmetries risk becoming structurally entrenched.

## 4 Policy strategies for narrowing global screening asymmetries

Addressing compounding asymmetries requires policy approaches that prioritise broad participation, realistic phasing, and operational feasibility. The objective is not to mandate immediate uniform, best-in-class screening globally, but to raise the global floor of responsible practice while enabling progressive strengthening over time.

A central risk in current policy discourse is inadvertent policy leapfrogging. As guidance increasingly reflects second-generation challenges such as AI-assisted design, functional screening, and benchmarking, expectations may implicitly assume levels of technical maturity that many providers have not yet reached. When advanced practices are framed as entry conditions rather than long-term aspirations, providers outside early-adopting jurisdictions may disengage entirely, reinforcing existing gaps.

### 4.1 A spectrum approach to adoption

**FIGURE 1:**
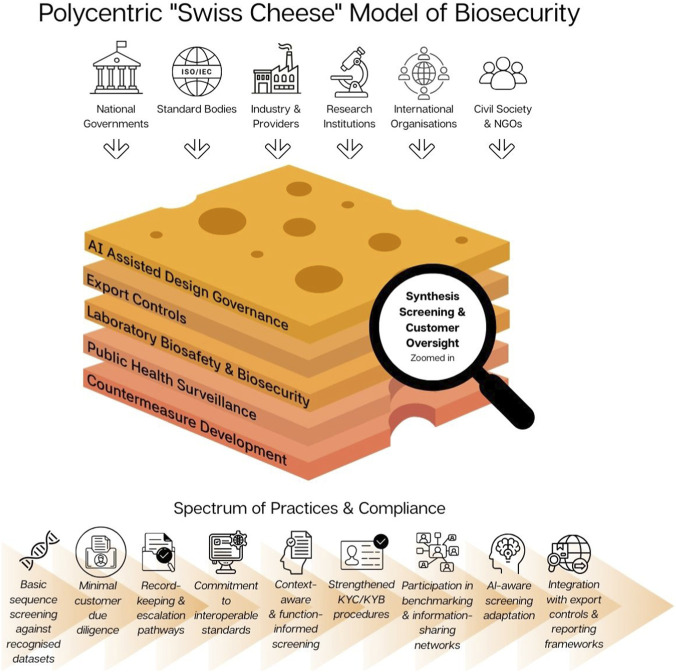
Polycentric swiss cheese model of biosecurity architecture and the spectrum of screening practices.

Rather than framing screening as a binary choice, policymakers should conceptualise adoption along a spectrum of participation (Figure 1). Within a layered biosecurity architecture, meaningful risk reduction can emerge from progressive movement along a continuum of practice. Figure 1 situates this spectrum within a polycentric Swiss cheese model, in which layers including AI design governance, sequence screening, customer due diligence, export controls, and institutional oversight are imperfect but partially overlapping. Each layer has inherent limitations, but incremental strengthening at any point reduces opportunities for regulatory arbitrage and weak-link vulnerabilities, even where advanced capabilities remain out of reach. Table 1 provides an expanded description of each spectrum component with recommended policy actions and progress indicators.

**TABLE 1:** Spectrum of screening practices and potential policy actions.

Spectrum component	Policy actions	Indicators of progress
Basic sequence screening against recognised datasets	Encourage or establish requirements (via governmental funding conditions or regulation) for providers to implement sequence screening against recognised, up-to-date datasets covering regulated and clearly high-risk agents. Provide access to low-cost or subsidised tools and reference sets; clarify minimum expectations for reporting matches	Proportion of domestic providers using some form of sequence screening; evidence of screening referenced in provider documentation; initial reporting of escalated sequences of concern
Harmonised appreciation of genetic sequences requiring customer legitimacy verification	Industry-wide or governmentally-mandated database of sequences subject to customer legitimacy verification requirements	Number of countries and regions that have adopted industry-wide or international standards for which genetic sequences should require customer legitimacy verification
Minimal viable customer due diligence (KYC/KYB lite)	Recommend baseline customer identity checks (e.g., verified institutional email, affiliation, delivery address), simple legitimacy screening, and basic internal red-flag criteria. Provide short, plain-language checklists and templates for providers with limited compliance capacity	Providers document basic KYC/KYB steps in internal SOPs; staff trained to recognise obvious red flags; orders declined or delayed due to customer concerns recorded and, where appropriate, notified to authorities
Record-keeping and internal escalation pathways	Define minimum expectations for retaining order, customer, and screening logs for a specified period; encourage designation of an internal biosecurity focal point; articulate simple escalation routes for suspicious orders and ambiguous screening hits	Documented escalation SOPs; proportion of providers with named biosecurity or compliance contact; evidence of escalation rather than silent rejection or fulfilment
Commitment to interoperable standards and reference frameworks	Promote voluntary alignment with international standards and frameworks (e.g., ISO 20688-2, IGSC guidance, national codes of practice) as reference points. Encourage common data formats, logging practices, and terminology to support future benchmarking and cross-border information sharing	References to recognised standards in national guidance and provider policies; convergence in terminology across providers; readiness to participate in cross-border pilots or audits
Context-aware and function-informed screening	Encourage providers to incorporate contextual information (sequence function, genomic neighbourhood, construct design) beyond identity matching. Support access to tools and expert networks that help interpret ambiguous hits	Evidence that providers adjust screening responses based on functional risk; documented cases where orders modified or refused due to functional concerns, not just identity matches
Strengthened KYC/KYB procedures	Support development of more robust verification for higher-risk orders (e.g., verification of licences, institutional authorisations, cross-checks with biosafety officers). Encourage risk-tiered approaches scaling scrutiny with sequence sensitivity	Increased use of enhanced verification for sequences of concern; coordination with institutional biosafety offices; reduction in unexplained high-risk orders from unverifiable entities
Participation in benchmarking and information-sharing networks	Facilitate provider participation in benchmarking and proficiency testing initiatives. Encourage voluntary reporting of suspicious patterns and support trusted, legally protected channels for sharing anonymised data on rejected sequences and circumvention attempts	Number of providers in benchmarking exercises; performance data showing improved detection over time; regular contributions to anonymised incident databases or consortia-level threat reports
AI-aware screening adaptation	Integrate awareness of AI-enabled sequence and protein design tools into guidance, including potential for low-identity yet functionally equivalent sequences. Encourage providers to update screening rulesets and engage with technical consortia on near-horizon threats	Guidelines and regulatory regimes explicitly reference AI-enabled design risks; periodic updates to screening algorithms documented; detection of atypical low-identity orders triggering additional scrutiny
Integration with export controls and reporting frameworks	Align screening expectations with export control regulations, biosafety rules, and relevant AI governance instruments. Clarify how suspicious orders and detected circumvention attempts should be reported into national or international mechanisms	Screening outcomes referenced in export control licensing; clear guidance linking screening to reporting mechanisms; documented cases where screening triggers regulatory investigation
Legal and procurement-backed enforcement	Link public procurement and funding to screening-aligned providers. In higher-capacity settings, establish legal requirements with proportionate sanctions. Consider certification schemes. Ensure enforcement is sequenced after credible entry pathways and support	Proportion of publicly funded projects requiring screening-compliant providers; proportionate enforcement actions; continued participation by smaller providers rather than exit from the regulated market.

At the entry end, providers might implement basic sequence screening against recognised datasets, feasible customer due diligence, responsible record-keeping, and clear escalation pathways. Further along the spectrum, providers may adopt context-aware screening, strengthened multi-pathway customer verification, participation in benchmarking networks, and adaptation to AI-enabled design risks. An illustrative example of this escalatory logic is the evolution of sequences-of-concern inclusion criteria: baseline screening under the earliest U.S. frameworks was limited to sequences uniquely found in regulated agents (ASPR, 2010; IGSC, 2009; IGSC, 2017); intermediate practice focuses on genes from regulated agents known to contribute to pathogenicity or toxicity (IGSC, 2024; DSIT, 2024); and the most advanced approaches seek to identify functionally equivalent sequences from non-regulated agents or AI-redesigned variants with low identity to known threats (ASPR, 2023a; NSTC, 2024; Wittmann et al., 2025). The policy objective should be to lower barriers to initial participation while maintaining credible minimum expectations and preserving incentives for progressive strengthening (OECD, 2024).

Importantly, the challenge is not solely technical. Advanced screening tools with low barriers to adoption now exist, yet a provider in sub-Saharan Africa or South Asia that screens in the absence of national guidance, funding conditions, or peer networks normalising participation does so in a vacuum, with no institutional recognition and no clear escalation pathway for suspicious orders. IBBIS’s mapping across more than 60 countries reveals a consistent pattern: jurisdictions without explicit synthesis biosecurity policy tend also to lack provider-level screening infrastructure, even where commercial synthesis is established (IBBIS, 2025a; IBBIS, 2025b). Policy absence actively undermines the operational rationale for adopting available tools. Capacity and policy constraints are mutually constitutive, and interventions targeting both dimensions simultaneously are likely to be substantially more effective than those treating them separately.

### 4.2 Operational mechanisms for accelerating convergence in practice

Translating this framing into practice requires complementary mechanisms such as.

#### 4.2.1 Demonstration and pilot initiatives

Targeted pilot projects in low and middle income or emerging synthesis markets can demonstrate what minimal viable screening looks like in context. Structured as public private partnerships, these pilots can test technical feasibility, identify regulatory ambiguities, and generate locally grounded reference models without requiring immediate legal harmonisation.

#### 4.2.2 Practical implementation guidance

High level standards and guidance should be complemented by provider facing materials that outline entry pathways and upgrade options in plain language. Templates for screening workflows, customer due diligence checklists, escalation procedures, and record keeping practices can reduce uncertainty and lower perceived barriers to entry.

#### 4.2.3 Shared technical infrastructure and benchmarking

International, multi stakeholder technical consortia focused on operationalising screening standards such as the DSSC and the SBRC provide mechanisms for translating abstract principles into interoperable practices. By developing shared definitions, reference datasets, and benchmarking approaches, such initiatives can reduce duplication, increase trust, and promote convergence without imposing uniform regulatory models.

#### 4.2.4 Incentive alignment and signalling

Funding agencies, procurement policies, and research collaborations can incorporate screening participation as a positive signal of responsible practice. Such measures need not require advanced capability; recognising baseline participation can help counteract the current dynamic in which responsible providers internalise compliance costs while competitors free ride on weaker standards.

Taken together, these mechanisms aim to make participation easier than abstention. By clarifying credible entry points, supporting incremental improvement, and aligning incentives with responsible practice, policymakers can reduce the structural forces that allow screening asymmetries to compound over time.

## 5 Discussion and implications

Current fragmentation in nucleic acid synthesis screening has direct implications for both deterrence and equity. In a globally networked supply chain, malicious actors do not need to defeat screening systems; they need only bypass them geographically. Partial adoption creates routing incentives, allowing high risk orders to flow toward less regulated providers. As AI-enabled design makes it easier to generate low-homology but functionally concerning sequences, these routing incentives increasingly intersect with technical blind spots, further amplifying the value of least-regulated nodes in the system. Under such conditions, the protective value of screening remains constrained by the least stringent nodes in the system.

This dynamic also generates economic distortions. Providers that invest in screening internalise compliance costs, while those operating without comparable safeguards may benefit from lower administrative burdens and faster turnaround times. The result is a perverse incentive structure in which responsible firms effectively subsidise global biosecurity while competitors free ride. Without broader participation, deterrence remains uneven and market pressures may further entrench asymmetries.

Importantly, in many regions outside the United States, New Zealand, and Europe, screening is not a contested policy idea; it is simply absent from active governance discourse. This suggests that the primary bottleneck is not always cost or technical capacity, but awareness, norm setting, and institutional clarity. Addressing asymmetries therefore requires engagement strategies that expand policy imagination as much as they expand infrastructure.

These realities reinforce the importance of embedding synthesis screening within a layered and polycentric biosecurity framework. Screening cannot eliminate misuse risk, but it can significantly raise the baseline level of friction and oversight when widely adopted. Its value depends on alignment with complementary mechanisms and on participation that is sufficiently broad to reduce weak link vulnerabilities.

Avoiding policy leapfrogging is therefore central to effective global strategy. Adoption pathways that recognise meaningful but incremental participation and lower entry barriers can prevent provider economies from being left behind while preserving incentives for progressive strengthening. A spectrum based approach allows jurisdictions to begin with feasible baseline measures and build capacity over time, rather than facing an implicit choice between full sophistication and non participation.

Neutral international conveners, such as the OECD and the World Health Organisation, may play a constructive role in facilitating baseline agreements and encouraging convergence across diverse regulatory environments. At the operational level, pilot projects in low and middle income contexts, supported through public private partnerships, can demonstrate minimal viable screening in practice and generate locally grounded models for expansion. In parallel, international multi stakeholder consortia devoted to improving and operationalising screening standards, such as DSSC and the SBRC, provide practical mechanisms for addressing shared implementation barriers and promoting interoperability.

As synthesis technologies continue to diffuse and AI enabled design tools accelerate biological innovation, uneven screening adoption is likely to magnify risk. The most urgent task is not to perfect screening within a small number of advanced jurisdictions, but to normalise baseline participation across the global biotechnology ecosystem. By lowering barriers to entry, aligning incentives, and reinforcing coordination mechanisms, policymakers can narrow asymmetries and strengthen collective deterrence while promoting a more equitable distribution of biosecurity responsibility.

### 5.1 Limitations

This perspective has several limitations. It draws on publicly available policy documents, international standards, and IBBIS mapping data rather than primary empirical research; the rapidly evolving policy landscape means that some developments may not be captured at the time of publication. The spectrum framework is conceptual and has not been empirically validated through case studies or pilot implementations. The analysis focuses primarily on commercial nucleic acid synthesis providers and does not systematically address risks associated with benchtop or academic synthesis, which represent a growing but distinct governance challenge. Finally, the discussion of LMIC contexts draws on available mapping data and published interviews but does not claim comprehensive regional coverage.

Polycentric Swiss cheese model of biosecurity governance and the spectrum of nucleic acid synthesis screening practices. The layered architecture illustrates how screening operates as one imperfect but essential layer alongside AI design governance, customer due diligence, export controls, institutional biosafety, public health surveillance, and countermeasure development. Multiple actors such as national governments, standards bodies, industry providers, research institutions, international organisations, and civil society, contribute complementary governance functions. The magnified “Synthesis Screening & Customer Oversight” layer shows the spectrum of practices from foundational measures (basic sequence screening and customer due diligence) through to advanced capabilities (context-aware and AI-assisted screening, interoperability with international standards, and integration with reporting frameworks).

## Data Availability

The original contributions presented in the study are included in the article/supplementary material, further inquiries can be directed to the corresponding author.
